# Were the sharp declines of dragonfly populations in the 1990s in Japan caused by fipronil and imidacloprid? An analysis of Hill’s causality for the case of *Sympetrum frequens*

**DOI:** 10.1007/s11356-018-3440-x

**Published:** 2018-10-20

**Authors:** Kosuke Nakanishi, Hiroyuki Yokomizo, Takehiko I. Hayashi

**Affiliations:** National Institute for Environmental Studies, Onogawa 16-2, Tsukuba, Ibaraki, 305-8506 Japan

**Keywords:** Agrochemicals, Neonicotinoid, Nursery box, Paddy fields, Pesticides, Phenylpyrazole, Rice fields, Odonata

## Abstract

Neonicotinoids and fipronil are the most widely used insecticides in the world. Previous studies showed that these compounds have high toxicity to a wide taxonomic range of non-target invertebrates. In rice cultivation, they are frequently used for nursery-box treatment of rice seedlings. The use of fipronil and neonicotinoid imidacloprid is suspected to be the main cause of population declines of red dragonflies, in particular *Sympetrum frequens*, because they have high lethal toxicity to dragonfly nymphs and the timing of the insecticides’ introduction in Japan (i.e., the late 1990s) overlapped with the sharp population declines. However, a causal link between application of these insecticides and population declines of the dragonflies remains unclear. Therefore, we estimated the amount of the insecticides applied for nursery-box treatment of rice seedlings and analyzed currently available information to evaluate the causality between fipronil and imidacloprid usage and population decline of *S*. *frequens* using Hill’s causality criteria. Based on our scoring of Hill’s nine criteria, the strongest lines of evidence were strength, plausibility, and coherence, whereas the weakest were temporality and biological gradient. We conclude that the use of these insecticides, particularly fipronil, was a major cause of the declines of *S*. *frequens* in Japan in the 1990s, with a high degree of certainty. The existing information and our analyses, however, do not allow us to exclude the possibility that some agronomic practices (e.g., midsummer drainage or crop rotation) that can severely limit the survival of aquatic nymphs also played a role in the dragonfly’s decline.

**Electronic supplementary material:**

The online version of this article (10.1007/s11356-018-3440-x) contains supplementary material, which is available to authorized users.

## Introduction

Neonicotinoids and the phenylpyrazole fipronil are the most widely used insecticides worldwide (Simon-Delso et al. 2015). They have systemic properties and are used to protect many crops through a variety of treatments, including seed coating, bathing, foliar spray, soil drench, and trunk injection (Jeschke et al. 2011). These insecticides are highly toxic to a wide range of invertebrates, including non-target organisms (reviewed by Pisa et al. 2015, 2017; Van Der Sluijs et al. 2015), although they have relatively low toxicity to humans.

In rice-producing countries, neonicotinoids and fipronil are frequently used in the cultivation of rice (*Oryza sativa* L.), which is one of the main crops in Asia. Worldwide, the total area of rice fields harvested in 2016 was approximately 1.9 billion ha (FAO 2017). Because 80% of the rice fields in Asia are irrigated under a monsoon climate (Kiritani 2000), flooded rice fields can provide habitats for a wide range of aquatic organisms and sustain biodiversity as an alternative to natural wetlands (Katayama et al. 2015; Lawler 2001; Natuhara 2013).

Dragonflies (Odonata) commonly inhabit rice fields, where they prey on insect pests and other aquatic organisms. Of the approximately 200 odonate species in Japan, 31 use rice fields (Uéda 1998). Among them, red dragonflies, *Sympetrum* spp. (Odonata: Libellulidae), and in particular *S*. *frequens*, are the most common species that use rice fields as reproductive sites (Uéda 1998).

Beginning in the 1990s, it was reported that populations of *S*. *frequens* and other *Sympetrum* species were rapidly and severely declining (to near extinction) in many regions of Japan (Fukui 2012; Futahashi 2012). The use of fipronil and the neonicotinoid imidacloprid is suspected as the main cause of these population declines, because the rapid declines occurred just after introduction of these systemic insecticides (Uéda and Jinguji 2013). In addition, experimental studies have indicated that fipronil and imidacloprid have high lethal toxicity to *Sympetrum* and other dragonfly species (Jinguji et al. 2013; Jinguji and Uéda 2015; Kasai et al. 2016). However, the relationship between the environmental dose of fipronil and imidacloprid in rice fields—the main habitat of *Sympetrum* dragonflies—and their population declines has never been systematically examined. The insecticides applied to nursery boxes containing rice seedlings are likely to have the most severe impact on *Sympetrum* populations because nymphs in the early developmental stage are exposed to relatively high concentrations of the insecticides released from rice seedlings just after transplanting (Thuyet et al. 2013; Sánchez-Bayo and Goka 2006). Data on the usage amount of the insecticides disaggregated by means of application are required to examine the relationship between use of the insecticides and dragonfly population declines, but, unfortunately, these data are not available. We thus have to estimate the amounts for each means of application (particularly those for nursery-box treatment of rice seedlings) to analyze the relationship between the insecticides and population declines of *S*. *frequens* in the 1990s.

In this study, we estimated the usage amounts of nine systemic insecticides, including fipronil and imidacloprid, applied to nursery boxes each year from 1989 to 2011. We then collected and examined currently available information to analyze the causal link between usage of fipronil and imidacloprid and the population declines of *S*. *frequens* in the 1990s according to Hill’s causality criteria (Hill 1965). These criteria were originally proposed as “viewpoints” to distinguish causal associations from non-causal ones by Sir Austin Bradford Hill (1965). They have been used as criteria for causal inference in epidemiological studies (Rothman et al. 2008) and in several ecotoxicological studies examining the relationships between imidacloprid concentrations and the abundance of aquatic macroinvertebrates (Van Dijk et al. 2013) and between neonicotinoid usage and the abundance of honey bees (Cresswell et al. 2012). Scoring based on Hill’s causality criteria can be helpful in evaluating causality based on insufficient experimental evidence (Cresswell et al. 2012). This procedure can also identify which criteria lack evidence as well as what further evidence is needed, although some of Hill’s causality criteria are difficult to apply to some environmental factors (e.g., the experimental evidence criterion for climate change). Our goal in this study was to evaluate causality between fipronil and imidacloprid usage and population declines of *S*. *frequens* in the 1990s in Japan. We also evaluated other potential factors to examine the specificity of the contribution from these insecticides and provide integrated scientific knowledge for conservation of this dragonfly.

## Materials and methods

### Dragonfly species

In this study, we focus on the red dragonfly, *S*. *frequens*, and several congeneric species, which inhabit mainly rice paddies in Japan. *Sympetrum frequens* is distributed throughout Japan and is common in Honshu and Hokkaido (Sugimura et al. 1999). This dragonfly also symbolizes rural life for the Japanese people (Jinguji and Uéda 2015).

**Fig. 1:**
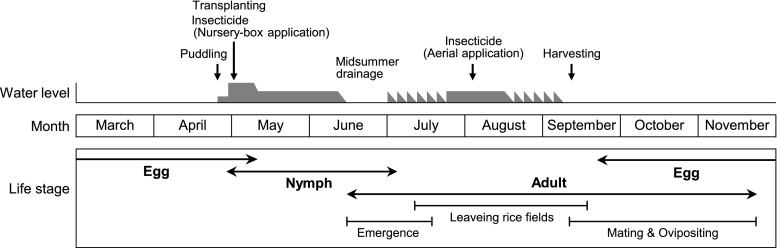
Annual life cycle of *Sympetrum frequens* and the timing of insecticide application and other main management practices in typical rice cultivation in central Japan. The times noted vary regionally to some extent

The life cycle of *S*. *frequens* and a brief schedule of typical cultivation management in rice fields are shown in Fig. 1. *Sympetrum frequens* is a univoltine insect. In late September, the adults come to drained rice fields after harvesting and lay eggs into puddles. The eggs hatch soon after the start of irrigation, around April. The nymphs grow while feeding on other invertebrates, such as microcrustaceans (water fleas and ostracods), midges, and mosquito larvae, and they emerge as adults in June and July. The newly emerged adults then migrate to mountainous areas far away from rice fields, and in late September they return to rice fields to start mating (Inoue and Tani 2010).

### Estimation of insecticide usage amounts

We estimated the usage amounts of nine systemic insecticides applied to nursery boxes of rice seedlings: the neonicotinoids imidacloprid, dinotefuran, clothianidin, and thiamethoxam; the phenylpyrazole fipronil; the carbamates benfuracarb and carbosulfan; the thiocarbamate cartap; and the anthranilic diamide chlorantraniliprole, which were commonly used around the study period in Japan.

We used the method of Yachi et al. (2016) for the estimation. First, we obtained the annual shipping amount for each insecticide product by prefecture from *Nouyaku*-*youran*, pesticide handbooks published by the Japan Plant Protection Association, from 1989 to 2011. Second, we estimated the fraction of usage of each insecticide product for nursery-box treatment of rice seedlings from the application list for each product. Third, we multiplied the shipping amount by the fraction of usage of each product and by the percentage content of the active ingredient. We summed these usage amounts to calculate the usage amount for each active ingredient of the insecticides in each prefecture and each year. From the data of estimated usage amount for each insecticide applied to nursery boxes, we calculated the annual usage ratio of each insecticide in each prefecture as follows (Yachi et al. 2017):

$$ {\displaystyle \begin{array}{c}\mathrm{Usage}\ \mathrm{ratio}\ \left(\%\right)=\frac{\mathrm{rice}-\mathrm{paddy}\ \mathrm{area}\ \mathrm{exposed}\ \mathrm{to}\ \mathrm{an}\ \mathrm{in}\mathrm{secticide}\ \left(\mathrm{ha}\right)}{\mathrm{total}\ \mathrm{rice}-\mathrm{paddy}\ \mathrm{acreage}\ \left(\mathrm{ha}\right)}\times 100\\ {}=\frac{\mathrm{usage}\ \left(\mathrm{g}\right)/\mathrm{standard}\ \mathrm{dose}\ \mathrm{of}\ \mathrm{in}\mathrm{secticide}\ \mathrm{in}\ \mathrm{rice}\ \mathrm{fields}\ \left(\mathrm{g}/\mathrm{ha}\right)}{\mathrm{total}\ \mathrm{rice}-\mathrm{paddy}\ \mathrm{acreage}\ \left(\mathrm{ha}\right)}\times 100\end{array}} $$

In conventional rice cultivation, the insecticides for nursery boxes are applied once a year, so the numerator represents the total area exposured to an insecticide. Therefore, the usage ratio represents the relative area exposed to the insecticide among all rice paddy fields in a prefecture. If an insecticide was used in all cultivated rice fields at the standard dose, the total usage ratio is calculated to be 100%. The total usage ratio for nursery boxes can be more than 100% when an insecticide usage amount applied to nursery boxes of rice seedlings was overestimated by the method of Yachi et al. (2016), when multiple insecticides were used in the same field, or when an insecticide was applied at more than the standard dose. The rice-paddy acreage data were obtained from the crop statistics of the Ministry of Agriculture, Forestry and Fisheries (2016a). We estimated the usage ratio in three prefectures, Toyama, Ishikawa, and Shizuoka, for which we know the timings of *S*. *frequens* population decline. The monitoring data of *S*. *frequens* in Toyama, Ishikawa, and Shizuoka were obtained from Futahashi (2012), Uéda (2012), and Fukui (2012), respectively.

### Analysis of association between insecticide usage and dragonfly abundance

We conducted a multiple linear regression analysis to examine the associations between the usage ratio of each systemic insecticide and the abundance of *S*. *frequens* in Toyama Prefecture, which has sufficient data for the analyses. (Annual abundance data were not available for many years during the census period in Ishikawa and Shizuoka Prefectures.) The response variable was the annual growth rate of the abundance of *S*. *frequens*, and the explanatory variable was the annual increase of the estimated usage ratio of each insecticide from 1993 to 2004, during the period of sharp decline of the dragonfly. As explanatory variables, we used only those insecticides with a usage ratio of at least 5% in any year during the period (i.e., imidacloprid, dinotefuran, fipronil, cartap, and carbosulfan). We defined the annual population growth rate as

$$ \mathrm{Growth}\ \mathrm{rate}=\frac{N_t-{N}_{t-1}}{N_{t-1}}, $$

where *N*_*t*_ denotes the abundance of the dragonfly in study year *t*. We selected the best-fit model based on Akaike’s information criterion (AIC) using the step AIC function in the MASS package (Venables and Ripley 2002) in the statistical software R version 3.5.1 (R Core Team 2018). The data for the analysis and the R code are given in Online Resources 1 and 2, respectively.

### Analysis based on Hill’s causality criteria

**Fig. 2:**
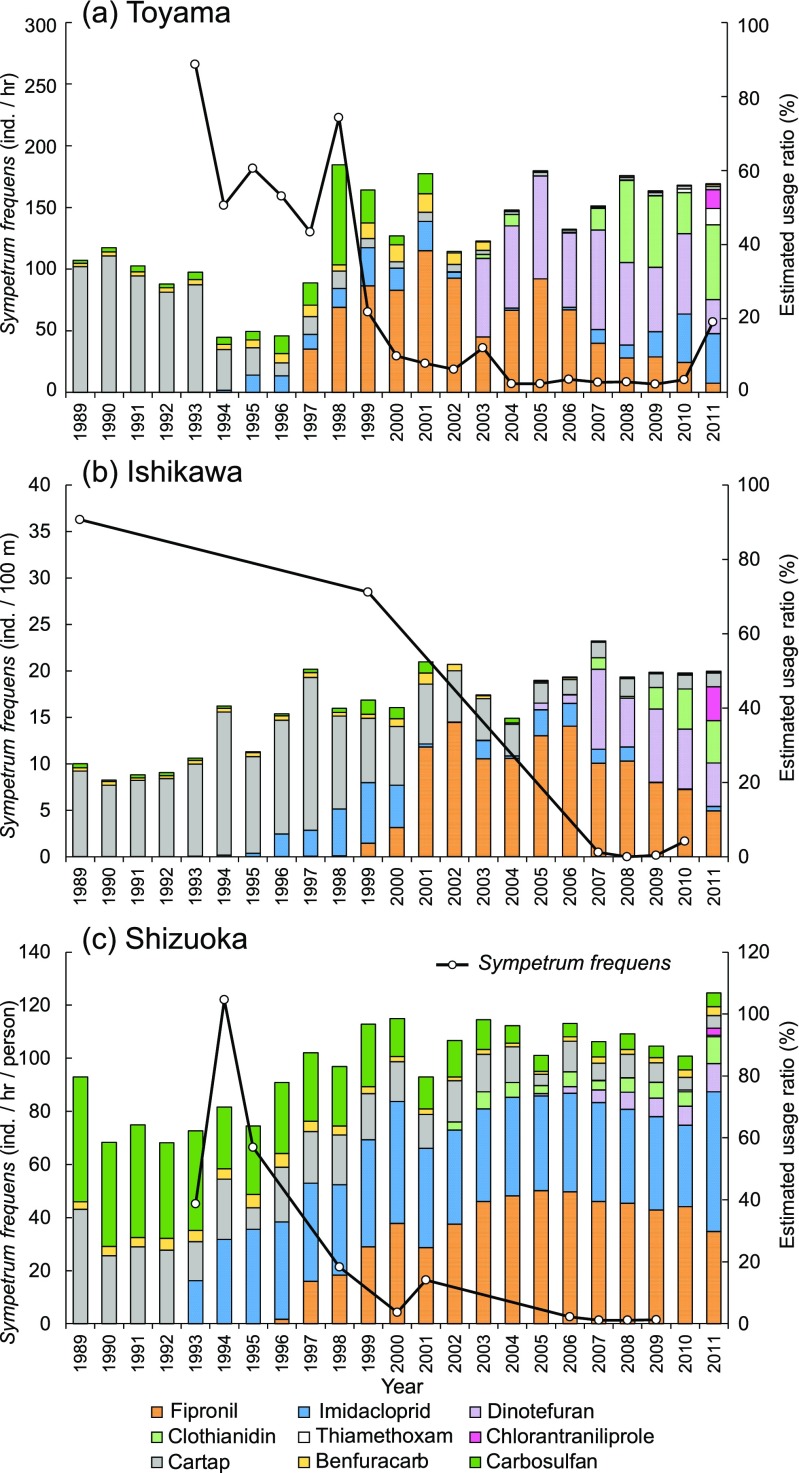
Estimated usage ratios of insecticides in nursery-box treatment of rice seedlings and density of *Sympetrum frequens* in **a** Toyama Prefecture, **b** Ishikawa Prefecture, and **c** Shizuoka Prefecture. Dragonfly data were from Futahashi (2012) for Toyama, Uéda (2012) for Ishikawa, and Fukui (2012) for Shizuoka. Because dragonfly census methods differed among these surveys, the density could not be standardized across prefectures

We analyzed the causal link between usage of fipronil and imidacloprid in nursery boxes and the population declines of *S*. *frequens* in the 1990s. The reason for focusing only on fipronil and imidacloprid is that our scope is the severe population declines of *S*. *frequens* in the 1990s in Japan. Fipronil and imidacloprid were the major insecticides used during that decade (Fig. 2), and application of these insecticides was proposed as the main cause for the species’ decline by Uéda and Jinguji (2013). Hill’s nine causality criteria (Hill 1965; Rothman and Greenland 2005) are (1) strength: a strong association is observed between a cause and an effect; (2) consistency: an association is observed repeatedly by different persons across different places, circumstances, and times; (3) specificity: a cause is specifically associated with an effect; (4) temporality: a cause precedes an effect; (5) biological gradient: a unidirectional dose–response relationship exists between a cause and an effect; (6) plausibility: an association can be explained by existing biological knowledge; (7) coherence: an association does not conflict with current natural history and biological knowledge; (8) experimental evidence: experimental results support an observed association; and (9) analogy: similar associations are known.

In our analysis based on Hill’s criteria, we considered the kinds of evidence relevant to a long-term (e.g., > 1 year) population declines of *S*. *frequens* under the environmental dose of fipronil or imidacloprid. For experimentally based associational criteria (i.e., strength, consistency, specificity, biological gradient, and experimental evidence), we considered both short-term and long-term experimental studies. In our evaluation, however, we placed more weight on those studies with endpoints relevant to long-term population decline in real paddy fields (e.g., more weight on emergence in a mesocosm than on 50% lethal concentration [LC_50_] from laboratory acute ecotoxicity tests), if both types of studies were available. For the consistency, specificity, and temporality criteria, field observation data and estimation of usage ratios of the insecticides were also considered. We cited experimental results on the other *Sympetrum* species inhabiting rice fields as well as *S*. *frequens.*

We scored the certainty of evidence at three levels for each criterion by the modified method of Cresswell et al. (2012). If the evidence likely to certainly supports (negates) the hypothesis, we give the criterion a score of + 3 (− 3). In the same way, if the evidence possibly to likely or slightly to possibly supports (negates) the hypothesis, we give scores of + 2 (− 2) and + 1 (− 1) to the criterion, respectively. If a criterion lacks evidence, it receives a score of 0.

## Results

### Association between insecticide usage and dragonfly abundance

Estimated usage ratios of insecticides for nursery-box application in the three prefectures are shown in Fig. 2. Before the introduction of neonicotinoids, cartap and carbosulfan were the dominant insecticides for nursery-box application. After the introduction of imidacloprid in 1993 and fipronil in 1996, their usage ratios rose rapidly, and these insecticides became dominant. In the mid-2000s, however, the usage ratios of dinotefuran and clothianidin increased, and they had overtaken those of imidacloprid and fipronil in Toyama and Ishikawa by the late 2000s. In Shizuoka, imidacloprid and fipronil consistently remained as the dominant insecticides since their first introduction.

The abundance of *S*. *frequens* in the three prefectures decreased sharply from the mid to late 1990s (Fig. 2), although the census methods and accuracy varied among locations. In the mid-2000s, very few individuals of this species were observed in the three regions. Our regression analysis showed that the best-fit model (i.e., that with the lowest AIC) includes four insecticides as explanatory variables: fipronil, imidacloprid, cartap, and carbosulfan (Table 1). Fipronil usage was negatively associated with dragonfly abundance (estimate = − 0.055, *P* = 0.008). Usage ratios of other insecticides showed positive associations with dragonfly abundance, but only that of carbosulfan (estimate = 0.050, *P* = 0.004) was statistically significant (Table 1). When conducting the same analysis using the sum of usage of all compounds in each insecticide class (i.e., fipronil, neonicotinoids, cartap, and carbamates) as explanatory variables, a similar result was obtained: fipronil usage and carbamate usage were negatively and positively associated with population growth, respectively (model 1 in Online Resource 2; scatter plots of each insecticide and insecticide class and population growth are shown as Fig. S1 in Online Resource 3). Note that fipronil and imidacloprid tended to be applied as alternatives to each other, especially in the 1990s. This means that a decrease in imidacloprid use was likely to be associated with an increase in fipronil use, and this could bias the regression coefficient of imidacloprid to the positive side.

**Table 1:** Summary of the best-fit model selected based on Akaike’s information criterion testing the statistical association between the annual increase of usage ratio of each insecticide and population growth of *Sympetrum frequens* from 1993 to 2004 in Toyama Prefecture

Variable	Estimate	SE	*t* value	*P* value
(Intercept)	0.077	0.108	0.715	0.502
Fipronil	− 0.055	0.014	− 3.874	0.008*
Imidacloprid	0.069	0.035	1.986	0.094
Cartap	0.033	0.018	1.811	0.120
Carbosulfan	0.050	0.011	4.633	0.004*

*P < 0.01

Next, we utilized the results reported above as evidence for the analysis based on Hill’s criteria.

### Analysis based on Hill’s causality criteria

**Fig. 3:**
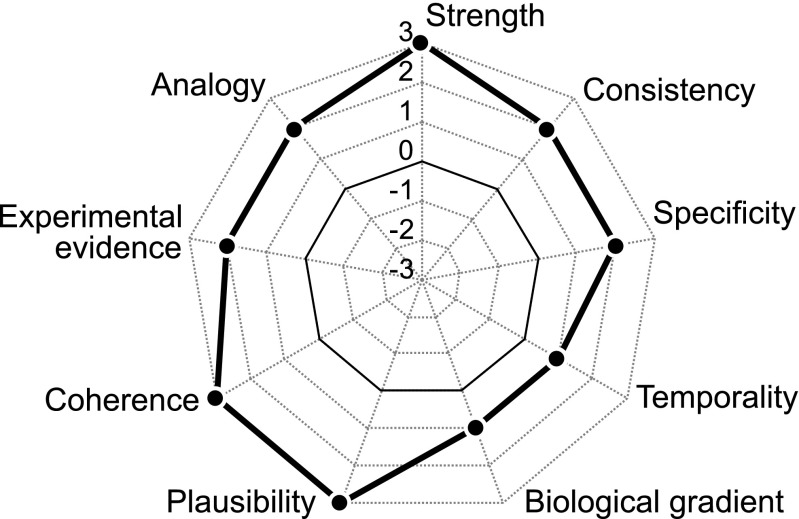
Scores of Hill’s nine causality criteria evaluating the association between fipronil and imidacloprid usage and population declines of *Sympetrum frequens* in Japan in the 1990s

All nine criteria received positive scores based on the analysis of reports in the literature and the association between insecticide usage and abundance of the dragonfly (Fig. 3). Strength, plausibility, and coherence had evidence that likely to certainly supported the hypothesis, whereas temporality and biological gradient had evidence that slightly to possibly supported it.

#### Strength

A general definition of strength in Hill’s criteria is “a strong association is observed between a cause and an effect.” Within the scope of this particular study, we defined strength as “a strong negative association between application of fipronil and imidacloprid and emergence of the *Sympetrum* species, which will directly drive population decline.” In this section, experimental study data are considered as evidence. As far as we know, no laboratory acute ecotoxicity test data using *S*. *frequens* have been published in publicly available form. Lysimetry experiments showed that fipronil had a serious effect on the emergence of *Sympetrum* species. In contrast to control treatments, application of fipronil at the recommended dose for commercial rice fields resulted in no *Sympetrum* adults’ emerging (Jinguji et al. 2009, 2013; Oyama and Kidokoro 2003; Suga et al. 2002). Jinguji and Uéda (2015) reported the same effect of imidacloprid on *S*. *frequens*. Such evidence of the severe effects of fipronil and imidacloprid on the dragonflies likely to certainly supported the hypothesis. Strength was therefore scored as + 3.

#### Consistency

A general definition of consistency in Hill’s criteria is “an association is observed repeatedly by different persons across different places, circumstances, and time.” Within the scope of this particular study, we define consistency as “a negative association between fipronil and imidacloprid and nymphal mortality or adult abundance of *Sympetrum* species observed repeatedly by different studies with various conditions (e.g., experimental types, places, times, or study groups).” In this section, experimental studies and field observation data are considered as evidence. We reviewed 21 experiments in 12 studies that examined the effects of fipronil and imidacloprid on *Sympetrum* species (Table 2). Among them, 17 experiments showed negative effects of the insecticides on survival rate and/or emergence rate of the dragonflies, although statistical significance levels differed among the experiments. In the other three experiments, no clear effects on *Sympetrum* species were detected.

**Table 2:** Studies on the effects of fipronil and imidacloprid on *Sympetrum* species

Insecticide	Species	Method^a^	System^b^	Application	Effect^c^	Effect details	Year^d^	Locality	Reference
Fipronil	*S*. *frequens*	TT	LYS	NBA^e^	LN	Increase mortality of nymphs; no adult emerged	2007	Akita, Japan	Jinguji et al. (2009)
	*S*. *frequens*	TT	ERF	NBA	LN	Decreased number of emerged adults	2008	Miyagi, Japan	Jinguji et al. (2009)
	*S*. *frequens*	TT	LYS	NBA	LN	Increased mortality of nymphs; no adult emerged	2010	Miyagi, Japan	Jinguji and Uéda (2015)
	*S*. *frequens*	TT	IV	10 mg/L^f^	LN	Lethal effect	2001, 2002	Saitama, Japan	Shimada et al. (2004)
	*S*. *frequens*, *S*. *infuscatum*	FO	LYS	NBA	NE	No effect detected	2010, 2011	Ibaraki, Japan	Hayasaka et al. (2013)
	*S*. *frequens*, *S*. *kunckeli*, *S*. *eroticum eroticum*, *S*. *infuscatum*, *S*. *darwinianum*, *S*. *annulata annulata*	FO	ERF	NBA	LN	Decreased number of emerged *S*. *frequens* and *S*. *kunckeli* adults	2008	Miyagi, Japan	Jinguji et al. (2013)
	*S*. *infuscatum*	TT	LYS	NBA	LN	Increased mortality of nymphs; no adult emerged	2008	Miyagi, Japan	Jinguji et al. (2013)
	*Sympetrum* spp.	FO	LYS	NBA	LN	Decreased number of nymphs; no adult emerged	2001	Iwate, Japan	Suga et al. (2002)
	*Sympetrum* spp. (including *S*. *darwinianum*)	FO	ERF	NBA	LN	Decreased number of nymphs	2002	Miyagi, Japan	Oyama and Kidokoro (2003)
	*Sympetrum* spp. (including *S*. *darwinianum*)	TT	LYS	NBA	LN	Increased mortality of nymphs; no adult emerged	2002	Miyagi, Japan	Oyama and Kidokoro (2003)
Imidacloprid	*S*. *frequens*	TT	LYS	NBA	SN	Increased mortality of nymphs; increased abnormal emergence	2007	Akita, Japan	Jinguji et al. (2009)
	*S*. *frequens*	TT	LYS	NBA	LN	Increased mortality of nymphs; no adult emerged	2010	Miyagi, Japan	Jinguji and Uéda (2015)
	*S*. *frequens*	TT	IV	100 mg/L^f^	SN	Lethal effect	2001, 2002	Saitama, Japan	Shimada et al. (2004)
	*S*. *frequens*, *S*. *infuscatum*, *S*. *croceolum*	FO	LYS	NBA	NE	No effect detected	2004	Ibaraki, Japan	Sánchez-Bayo and Goka (2006)
	*S*. *frequens*, *S*. *infuscatum*	FO	LYS	NBA	NE	No effect detected	2010, 2011	Ibaraki, Japan	Hayasaka et al. (2013)
	*S*. *infuscatum*	TT	LYS	NBA	LN	Increased mortality of nymphs	2008	Miyagi, Japan	Jinguji et al. (2013)
	*Sympetrum* spp.	TT	ERF	NBA	NE	No effect detected	2007	Tokyo, Japan	Motobayashi et al. (2012)
	*Sympetrum* spp. (including *S*. *darwinianum*)	FO	ERF	NBA	SN	Decreased number of nymphs	2002	Miyagi, Japan	Oyama and Kidokoro (2003)
	*Sympetrum* spp. (including *S*. *darwinianum*)	TT	LYS	NBA	SN	Increased mortality of nymphs	2002	Miyagi, Japan	Oyama and Kidokoro (2003)
	*Sympetrum* spp. (including *S*. *frequens*, *S*. *infuscatum*, *S*. *pedemontanum elatum*)	FO	ERF	NBA	SN	Decreased number of nymphs; reduced adult emergence	1998	Akita, Japan	Konno (1998)
Neonicotinoids and fipronil (unidentified)	*Sympetrum* spp. (including *S*. *frequens*, *S*. *darwinianum*, *S*. *infuscatum*)	FO	CRF	NBA	SN	Decreased number of emerged adults	2012	Niigata, Japan	Aoda et al. (2013)

^a^*TT* toxicity test, *FO* field observation

^b^*IV* in vitro, *LYS* lysimeter, *ERF* experimental rice field, *CRF* commercial rice field

^c^*LN* large negative effects, *SN* small negative effects, *NE* no effect detected

^d^Study year or the year of publication

^e^Nursery-box application at the recommended dose for commercial rice fields

^f^Concentration of significant lethal effect

Sharp declines in *S*. *frequens* abundance from the 1990s were observed in the three target prefectures in Japan (Fukui 2012; Futahashi 2012; Uéda 2012). We detected a statistically significant negative association between fipronil usage and abundance in Toyama Prefecture (Table 1). In the other regions, however, we could not statistically examine the association because we lacked sufficient long-term time series data on the species’ population dynamics.

We regarded the evidence as possibly to likely supporting the hypothesis. Consistency was therefore scored as + 2.

#### Specificity

A general definition of specificity in Hill’s criteria is “a cause is specifically associated with an effect.” Within the scope of this particular study, we define specificity as “the declines in *S*. *frequens* in the 1990s was specifically associated with fipronil and imidacloprid use, not with other factors.” In this section, experimental studies and field observation data of the species and studies on the effects of agronomic and environmental factors are considered as evidence. Among the nine systemic insecticides examined, only fipronil had a statistically significant negative relationship with abundance of the dragonfly (Table 1). Suga et al. (2002) have reported that the herbicides mefenacet and pyrazosulfuron-ethyl and the fungicide carpropamid had no effects on nymphal mortality and emergence rate of *Sympetrum* species, whereas fipronil decreased the number of nymphs and emergence rate. Shimada et al. (2004) also observed no effects of three herbicides and three fungicides on nymphal mortality of *S*. *frequens*. Field surveys of the number of *Sympetrum* exuviae also indicated no negative effects of herbicides (Aoda et al. 2013). In contrast, the report by Konno (2000) that the dragonfly emergence rate in experimental rice fields to which herbicides were applied was lower than in control fields indicates an indirect effect of herbicides. Shimada et al. (2004) reported strong lethal effects on *S*. *frequens* of carbosulfan, benfuracarb, cartap, imidacloprid, and fipronil applied at the recommended doses for commercial rice fields. Jinguji and Uéda (2015) reported that cartap delayed adult emergence of *S*. *frequens*, although it had no significant lethal effects compared with the control treatment. Other studies showed that pyrethroid, organochlorine, and organophosphate insecticides had high toxicity to nymphs of dragonflies (Fairchild et al. 1992; Ishida and Murata 1992; Suhling et al. 2000). However, these insecticides with high toxicity to dragonflies would not be a major cause of the declines of *S*. *frequens* in the 1990s, because these older insecticides had been used before the 1990s and did not cause severe population declines in dragonflies.

In conventional rice cultivation in Japan, midsummer drainage is commonly conducted in paddy fields. The paddy water is drained for a period of 7–10 days at around 40 days after transplanting (Fig. 1). Because dragonfly nymphs are aquatic, such drainage can eradicate them if their habitat is completely dried for a few days before their emergence. A negative effect of midsummer drainage on dragonfly emergence has been reported previously (Aoda et al. 2013; Yajima et al. 2002). Aoda et al. (2013) have reported that the number of adult *Sympetrum* that emerge in rice fields with midsummer drainage decreases to about 20% of those without midsummer drainage. The impact of midsummer drainage could have been enhanced by farmland consolidation and modernization, which increased the acreage of well-drained paddies. However, the rate of increase in consolidated farmland in Japan had slowed by the early 1990s, a few years before the sharp decline of the dragonfly populations (Ministry of Agriculture, Forestry and Fisheries 2016b). Crop rotation, which has been conducted as part of the rice acreage reduction policy of the Japanese government since the 1970s, may also have a negative impact on dragonfly abundance in some areas. In this crop rotation system, conversion of rice paddies to dry fields to cultivate crops such as wheat and soybean in the following year destroys the eggs of dragonflies laid in the rice paddies.

Climate change is another potential factor underlying the population declines of dragonflies. *Sympetrum frequens* adults migrate to mountains far away from rice fields during summer (Inoue and Tani 2010). Because of this behavior, Uéda (1988) hypothesized that the species must spend the summer season in a cool place with a mean temperature below approximately 23 °C. If the mean temperature rises due to climate change, the possible decrease or complete disappearance of suitable summer habitat for the dragonfly would then theoretically lead to a population decline. According to the Japan Meteorological Agency (2018), however, there has been no marked change in the mean summer temperature from the late 1990s to the present in Toyama Prefecture, although data in mountainous areas are not available. Precipitation can also affect abundance of *Sympetrum* species, particulary during midsummer drainage, because rain water can prevent rice fields from being dried well. Likewise, however, no systematic change of precipitation has been observed in Toyama Prefecture during the period of sharp population decline (Japan Meteorological Agency 2018).

Among the pesticides used around 2000 in Japan, fipronil and imidacloprid are comparatively highly toxic to nymphs of *Sympetrum*, which supports the hypothesis. There is no strong evidence that the use of other agrochemicals and climate change could be major causes of the declines of *S*. *frequens* in the 1990s. However, we cannot exclude the possibility that impacts of some agronomic factors (e.g., midsummer drainage, consolidation, and crop rotation) were major causes of the declines. Note that the possibility that agronomic factors were major causes of the severe declines is not mutually exclusive with the possibility that fipronil and imidacloprid were major causes. Population declines in different areas of Japan could be caused by different factors, and these factors also could cause severe population declines via their joint effects. Thus, we considered that this evidence possibly to likely supported the hypothesis that fipronil and imidacloprid specifically caused the *S*. *frequens* population declines in the 1990s. Specificity was therefore scored as + 2.

#### Temporality

A general definition of temporality in Hill’s criteria is “a cause precedes an effect.” Within the scope of this particular study, we define temporality as “available time series data of the declines statistically support the causality between the usage of fipronil and imidacloprid and the decrease of the abundance of *S*. *frequens*.” Sharp declines of *S*. *frequens* populations followed the introduction of imidacloprid and/or fipronil in the three prefectures (Fig. 2). The significant negative association between the annual increase of the usage ratio of fipronil and the population growth rate of *S*. *frequens* (Table 1) supported the hypothesis that fipronil and imidacloprid caused the population decline in the 1990s. However, this regression analysis did not provide strong evidence. The beginning of the population decline before the introduction of fipronil in Toyama Prefecture indicated that other factors affected the population dynamics as well. The population dynamics in a particular year can be greatly affected by many year-specific conditions, such as meteorological (e.g., temperature and precipitation) or biological (e.g., increase in competitive species) factors. In order to perform a causal temporal analysis between the insecticide usage and population growth, iterative data from multiple regions are needed to remove confounding factors. With such iterative data, we could separately examine the effect of study year and that of each insecticide by using alternative statistical models. Note that appropriate statistical procedures (e.g., proper control for confounding factors) are crucial for causative interpretation of time series data, because regression analysis of time series and field observation data are prone to result in spurious correlation. In addition, we only have data from one area (i.e., available data represent only one independent sample in terms of area variability), but the population decline in the 1990s occurred in many areas across Japan. This limits the statistical stability and generalization of our analysis. If we had series of numerical data to control for potential confounding factors and data from many prefectures, the score might be higher. However, no such data were available for the analysis. Based on these considerations, we regarded the evidence as slightly to possibly supporting the hypothesis. Temporality was therefore scored as + 1.

#### Biological gradient

A general definition of biological gradient in Hill’s criteria is “a unidirectional dose–response relationship exists between a cause and an effect.” Within the scope of this particular study, we define biological gradient as “a unidirectional negative dose–response relationship exists between concentrations of fipronil and imidacloprid and mortality of nymphs or emergence and adult abundance of *Sympetrum* species.” Shimada et al. (2004) conducted toxicity tests of fipronil and imidacloprid at three concentrations and have reported that there are gradients of the lethal dose to *S*. *frequens*. We could not find any other studies that show a relationship between insecticide dose and toxicity to the dragonflies. Toxicity values (e.g., 50% lethal concentration, LC_50_) of insecticides to *Sympetrum* species are also lacking, except for the LC_50_ of the neonicotinoid thiacloprid to *Sympetrum striolatum*, a common species in Europe (LC_50_ = 31.2 μg/L; Beketov and Liess 2008).

Field research has shown that concentrations of fipronil (Mize et al. 2008) and imidacloprid (Van Dijk et al. 2013) in environmental water bodies had significant negative effects on the abundance of a wide taxonomic range of aquatic macroinvertebrates. However, we could not examine the relationship between the insecticide concentrations and *S*. *frequens* abundance, because quantitative data on population dynamics of the dragonfly were available only in Toyama Prefecture. Based on these considerations, we concluded that this evidence slightly to possibly supports the hypothesis. Biological gradient was therefore scored as + 1.

#### Plausibility

A general definition of plausibility in Hill’s criteria is “an association can be explained by existing biological knowledge.” Within the scope of this particular study, we define plausibility as “a causative association between fipronil and imidacloprid application and population declines of *S*. *frequens* can be explained by existing biological and ecotoxicological knowledge.” A causative association between fipronil and imidacloprid application and population decline of *S*. *frequens* is biologically plausible for the following reasons. First, the hatching of the eggs of *S*. *frequens* around the time when insecticide-treated rice seedlings are transplanted (Fig. 1) (Inoue and Tani 2010) implies that nymphs will be exposed to highly concentrated systemic insecticides during this important phase of development. Second, evidence indicates that fipronil and imidacloprid have lethal toxicity to nymphs of *S*. *frequens* when applied at the dose recommended for commercial rice fields (Table 2). Notably, with fipronil treatment, no adults emerged in some experiments (Jinguji et al. 2009; Jinguji and Uéda 2015). Third, because *S*. *frequens* reproduces mostly in rice fields (Inoue and Tani 2010; Sugimura et al. 1999), most populations are subject to the effects of these insecticides.

From the perspective of the modes of action of fipronil and imidacloprid, their causality among insecticides is plausible. Unlike the other insecticides, fipronil and neonicotinoids produce delayed and chronic mortality due to persistence of their toxic metabolites and the delayed mortality they produce (Beketov and Liess 2008; Rondeau et al. 2014).Therefore, fipronil and imidacloprid can eliminate the nymphs of *S*. *frequens* and prevent their emergence as adults, which could lead to a population decline in a single season.

From these considerations, we regarded that this evidence likely to certainly supports the hypothesis. Plausibility was therefore scored as + 3.

#### Coherence

A general definition of coherence in Hill’s criteria is “an association does not conflict with current natural history and biological knowledge.” Within the scope of this particular study, we define coherence as “an association between fipronil and imidacloprid and population declines of *S*. *frequens* does not conflict with present substantive knowledge about the impact of these insecticides on various other invertebrates.” Neonicotinoids and fipronil have lethal toxicity to various non-target taxonomic groups, with especially serious effects on invertebrates (Beketov and Liess 2008; Pisa et al. 2015, 2017) and rice field communities in particular (Hayasaka et al. 2012; Sánchez-Bayo et al. 2016). Previous research has shown that odonate species in rice fields other than *Sympetrum* decrease with application of fipronil (Hayasaka et al. 2012, 2013; Kasai et al. 2016) or imidacloprid (Hayasaka et al. 2012, 2013; Sánchez-Bayo and Goka 2006). Many previous studies have indicated that neonicotinoid usage is negatively associated with declines of the honey bee (*Apis mellifera* L.) and wild bees (e.g., Henry et al. 2012; Rundlöf et al. 2015; Woodcock et al. 2016, 2017). For these reasons, the hypothesis that *S*. *frequens* declined due to application of fipronil and imidacloprid does not conflict with established knowledge. Coherence was therefore scored as + 3.

#### Experimental evidence

A general definition of experimental evidence in Hill’s criteria is “experimental results support an observed association.” Within the scope of this particular study, we define experimental evidence as “experimental results support the association between fipronil and imidacloprid and population declines of *S*. *frequens*.” We checked if previous experimental studies revealed any effect of fipronil and imidacloprid (or other neonicotinoids) at the environmental dose in rice fields on the abundance and long-term (e.g., > 1 year) population declines of *S*. *frequens* and closely related *Sympetrum* species. Almost all the experiments conducted in the laboratory, experimental fields, and commercial rice fields showed a negative association between fipronil and imidacloprid usage and *Sympetrum* abundance (Table 2). However, there is no available experimental evidence yet regarding the association between insecticide usage and long-term (e.g., > 1 year) population declines of dragonflies. Thus, we considered that experimental evidence possibly to likely supports the hypothesis. Experimental evidence was therefore scored as + 2.

#### Analogy

A general definition of analogy in Hill’s criteria is “similar associations are known.” Within the scope of this particular study, we define analogy as “associations between fipronil and neonicotinoids or other insecticides and long-term (e.g., > 1 year) population declines of wild organisms are known.” A similar association has been shown in butterflies in the same time period; Forister et al. (2016) have detected a negative association between the size of butterfly populations and neonicotinoid application. There are similar examples of associations between other insecticides and population declines in vertebrates. Davidson (2004) showed that amphibian population declines were strongly associated with the historical usage of pesticides such as organophosphates and carbamates. Newton and Wyllie (1992) reported that a population of sparrowhawk (*Accipiter nisus* L.), which had declined after the introduction of organochlorine pesticides such as DDT in the 1950s, recovered as the residues of aldrin, dieldrin, and DDT in the environment decreased. However, the pathways of exposure are different from the case of *S*. *frequens* in Japan. We regarded this evidence as possibly to likely supporting the hypothesis. Analogy was therefore scored as + 2.

## Discussion

All of Hill’s causality criteria were scored as positive values. The strongest lines of evidence were three theoretical criteria, strength, plausibility, and coherence, whereas the weakest were temporality and biological gradient. Based on this evaluation of the association between insecticide usage and dragonfly abundance, we conclude that the use of these insecticides, particularly fipronil, was the main cause for the declines of *Sympetrum* populations in Japan in the 1990s, with a high degree of certainty. The existing information and our analyses, however, do not allow us to exclude the possibility that some agronomic practices (e.g., midsummer drainage, consolidation, and crop rotation) that can severely limit the survival of aquatic nymphs also contributed to the declines of *S*. *frequens*. Here, we discuss the weakness of the lines of evidence that could not support the hypothesis based on currently available information, as well as the need for additional research on this topic in the future.

Among Hill’s criteria, temporality and biological gradient had the weakest evidence. Recent population dynamics of *S*. *frequens* in Toyama Prefecture was significantly associated with the usage ratio of fipronil for nursery-box application (Table 1; Fig. 2a). However, the species’ populations showed large fluctuations that could not be accounted for by fipronil and neonicotinoids, especially before introduction of the insecticides. These may be natural fluctuations driven by meteorological factors, which are likely major confounding factors that can result in large biases in our regression analysis estimates. Because replicates of quantitative data of the dragonfly in different locations and data of insecticide usage at a more local scale were not available, we were unable to exclude the potential confounding effects of these factors and thus could not perform a causal temporal analysis with a sufficient level of certainty.

Many agricultural factors besides fipronil and imidacloprid usage could also affect population declines of the dragonflies in rice fields, such as fertilizers and various cultivation management methods that depend on rice variety, and consolidation of rice fields. The rate of increase in farmland consolidation, which could cause marked changes in dragonfly habitat, was reduced by the early 1990s in Japan (Ministry of Agriculture, Forestry and Fisheries 2016b), but an array of pesticides, fertilizers, and rice varieties have been introduced since the 1990s. These factors are also potential confounders and must be assessed quantitatively as we examine the specificity of fipronil and imidacloprid as the cause for the population declines in the 1990s. However, few quantitative studies have examined the effects of these agricultural factors on dragonfly population dynamics.

In this study, we considered fipronil and imidacloprid together as we evaluated whether use of the insecticides caused the population declines of *S*. *frequens*. Based on experimental evidence and analysis of the association between insecticide usage and dragonfly abundance, it appears that fipronil has a greater effect on the population declines than imidacloprid (Tables 1 and 2). This may result not only from the high toxicity of fipronil but also the toxicity of its metabolites (i.e., degradation products). Fipronil-sulfide and fipronil-sulfone, two metabolites of fipronil, are more toxic to aquatic macroinvertebrates than fipronil (Maul et al. 2008; Weston and Lydy 2014). In addition, residuals of fipronil in paddy soil are more persistent than those of imidacloprid (Hayasaka et al. 2012).

In addition to direct lethal effects, fipronil and imidacloprid may also have indirect effects on *S*. *frequens* population dynamics that are mediated by the food web. Community-level impacts of fipronil and imidacloprid on paddy aquatic organisms have been reported (Hayasaka et al. 2012; Kasai et al. 2016; Kobashi et al. 2017). With decreases in the abundance of prey organisms, such as cladoceran and chironomid larvae, mortality of the dragonfly nymphs could increase due to starvation. Declines in prey abundance may retard growth and result in defective development of dragonfly nymphs (Jinguji and Uéda 2015). It is difficult to predict the community-level impacts of the insecticides in the field, because community composition differs among locations. Kobashi et al. (2017) noted that the numbers of chironomid larvae and *Crocothemis servilia mariannae* nymphs increased in paddy mesocosms treated with dinotefuran. These increases were considered to be an indirect effect of reduced competition with other species decreased by the insecticide.

Most ecotoxicity studies of aquatic insects have focused on their aquatic stages, so there is a knowledge gap regarding the terrestrial life stage (Rasmussen et al. 2017). Therefore, we might also have to consider adult stages of the dragonflies in the terrestrial environment, although *S*. *frequens* adults generally inhabit mountains far away from rice fields during the aerial application of fipronil and imidacloprid to rice plants in summer. The adults may be indirectly exposed to the insecticides through ingestion of contaminated prey (Walker et al. 2007), such as adult chironomids, and the decline in adult chironomid populations due to insecticide application may also affect the abundance of predatory adult dragonflies.

Additional research can further help to identify the causal link between fipronil and imidacloprid and population declines of *Sympetrum* dragonflies. Although it is difficult to obtain historic quantitative data of the dragonfly populations other than the examples cited here, monitoring the current status of dragonfly populations in different locations where the usage ratios of the insecticides differ would help to clarify the causal link between insecticide usage and population declines. Moreover, conducting laboratory ecotoxicity tests of fipronil and imidacloprid to *S*. *frequens* and related species would help us to assess the toxicity of these insecticides using standard toxicity indices like LC_50_ values. It is also important to bridge the knowledge gap among laboratory tests, mesocosm experiments, and field studies, because a delayed or sublethal effect (e.g., decreased feeding activity) leading to population declines of dragonflies may be hardly detectable in short-term laboratory experiments, for example. The estimated usage ratio of each insecticide was relatively low, and the total usage ratios were 60% at most in Toyama and Ishikawa Prefectures (Fig. 2), implying that at least 40% of the paddy fields were not exposed to the nine insecticides. Because almost all reproductive sites of *S*. *frequens* are within rice fields in Japan, this conclusion might quantitatively contradict the hypothesis that insecticides caused the severe decline of the dragonfly in the 1990s in these regions. In other words, some other factors also likely contributed to the severe decrease (e.g., a 97% decline from 1993 to 2004 in Toyama Prefecture; Fig. 2a) while 40% of the paddy fields were not exposed to the insecticides in these regions. Evaluating the impacts of individual agronomic practices by experimental studies and/or field observation would provide information about how *S*. *frequens* effectively utilize paddy fields not exposed to the insecticides. Such data would allow us to infer the absolute effect of fipronil and imidacloprid on the population declines of *Sympetrum* dragonflies and to develop effective conservation strategies (e.g., choice of harmless insecticides and the creation of mitigation sites) to recover the populations. If the causal link between the insecticide usage and the abundance of dragonflies is clarified, we may be able to predict future population dynamics using demographic models of *Sympetrum* population growth involving these environmental factors.

In this study, we scored each of Hill’s nine criteria to evaluate the causality of fipronil and imidacloprid usage in *S*. *frequens* population declines in the 1990s. In general, there are two possible reasons why some criteria were scored as low positive values: (1) a lack of evidence when there is little available information, and (2) a conflict between positive and negative evidence. Most criteria in the current evaluation fit the first situation. In addition, some lines of evidence, such as those from ecotoxicological experiments, were considered for multiple criteria (e.g., strength, consistency, and experimental evidence), which means that Hill’s criteria are not independent. Therefore, we cannot judge the absolute degree of causality based on each criterion, and we cannot use total (i.e., summed) scores of the different criteria for any purpose. Instead, by clarifying the definitions and evidence to be considered for each criterion, this analysis has enabled us to identify what kinds of evidence are available or lacking and what further research is needed. Our analysis based on Hill’s criteria also provides a summary for developing conservation policy to mitigate population declines of *S*. *frequens* and congeneric dragonflies.

## Electronic supplementary material

ESM 1 (csv 1.80 kb)

ESM 2 (docx 12.0 kb)

ESM 3 (docx 96.8 kb)
