# Supplementary material for: Were the sharp declines of dragonfly populations in the 1990s in Japan caused by fipronil and imidacloprid? An analysis of Hill’s causality for the case of Sympetrum frequens
Source: Environ Sci Pollut Res Int. 2018 Oct 20;25(35):35352–64. doi: 10.1007/s11356-018-3440-x (PMC6280840; doi:10.1007/s11356-018-3440-x)
Supplement: Supplementary file 2 — (docx 12.0 kb) [file 11356_2018_3440_MOESM2_ESM.docx]

Online Resource 2

Nakanishi et al.

September 18, 2018

Article title: Were the sharp declines of dragonfly populations in the 1990s in Japan caused by fipronil and imidacloprid? An analysis of Hill’s causality for the case of Sympetrum frequens

Journal name: Environmental Science and Pollution Research

Author names: Kosuke Nakanishi, Hiroyuki Yokomizo, Takehiko I. Hayashi

Affiliation and e-mail address of the corresponding author: National Institute for Environmental Studies, Onogawa 16-2, Tsukuba, Ibaraki 305-8506, Japan; [nakanishi.kosuke@nies.go.jp](mailto:nakanishi.kosuke@nies.go.jp)

### Calculate annual growth rate of Sympetrum frequens and annual increase of the estimated usage ratio of nine insecticides

library(dplyr)

##
## Attaching package: 'dplyr'

## The following objects are masked from 'package:stats':
##
## filter, lag

## The following objects are masked from 'package:base':
##
## intersect, setdiff, setequal, union

library(tibble)
library(MASS)

##
## Attaching package: 'MASS'

## The following object is masked from 'package:dplyr':
##
## select

d <- read.csv("ESM_1.csv", header = TRUE)
d2 <- data_frame(Sympetrum.growth =(d$Sympetrum[2:19]-d$Sympetrum[1:18])/d$Sympetrum[1:18]) %>% mutate(Fipro.dif=diff(d$Fipronil),Imida.dif=diff(d$Imidacloprid), Dinotefu.dif=diff(d$Dinotefuran), Clothia.dif=diff(d$Clothianidin), Thiametho.dif=diff(d$Thiamethoxam), Chloran.dif=diff(d$Chlorantraniliprole), Cartap.dif=diff(d$Cartap), Benfura.dif=diff(d$Benfuracarb), Carbosu.dif=diff(d$Carbosulfan)) %>% add_column(Year=1994:2011)

### Multiple linear regression analysis with a model selection using the stepAIC function

#### Model 1: by individual insecticide

model_1 <- glm(Sympetrum.growth~Fipro.dif + Imida.dif + Dinotefu.dif + Cartap.dif + Carbosu.dif, family=gaussian, data = d2[d2$Year<=2004,])
summary(stepAIC(model_1))

## Start: AIC=10.44
## Sympetrum.growth ~ Fipro.dif + Imida.dif + Dinotefu.dif + Cartap.dif +
## Carbosu.dif
##
## Df Deviance AIC
## - Dinotefu.dif 1 0.52202 9.6892
## <none> 0.46600 10.4405
## - Cartap.dif 1 0.59749 11.1746
## - Imida.dif 1 0.63905 11.9144
## - Fipro.dif 1 0.71430 13.1388
## - Carbosu.dif 1 1.80960 23.3640
##
## Step: AIC=9.69
## Sympetrum.growth ~ Fipro.dif + Imida.dif + Cartap.dif + Carbosu.dif
##
## Df Deviance AIC
## <none> 0.52202 9.6892
## - Cartap.dif 1 0.80735 12.4858
## - Imida.dif 1 0.86521 13.2471
## - Fipro.dif 1 1.82747 21.4720
## - Carbosu.dif 1 2.38967 24.4225

##
## Call:
## glm(formula = Sympetrum.growth ~ Fipro.dif + Imida.dif + Cartap.dif +
## Carbosu.dif, family = gaussian, data = d2[d2$Year <= 2004,
## ])
##
## Deviance Residuals:
## Min 1Q Median 3Q Max
## -0.55082 -0.06155 0.02100 0.14489 0.32430
##
## Coefficients:
## Estimate Std. Error t value Pr(>|t|)
## (Intercept) 0.07722 0.10806 0.715 0.50170
## Fipro.dif -0.05515 0.01424 -3.874 0.00823 **
## Imida.dif 0.06938 0.03493 1.986 0.09422 .
## Cartap.dif 0.03318 0.01832 1.811 0.12012
## Carbosu.dif 0.04999 0.01079 4.633 0.00357 **
## ---
## Signif. codes: 0 '***' 0.001 '**' 0.01 '*' 0.05 '.' 0.1 ' ' 1
##
## (Dispersion parameter for gaussian family taken to be 0.08700262)
##
## Null deviance: 3.00317 on 10 degrees of freedom
## Residual deviance: 0.52202 on 6 degrees of freedom
## AIC: 9.6892
##
## Number of Fisher Scoring iterations: 2

#### Model 2: by insecticide class

d3 <- d %>%
 mutate(Neonics=Imidacloprid+Dinotefuran+Clothianidin+Thiamethoxam) %>%
 mutate(Carbamates=Benfuracarb+Carbosulfan)

d4 <- data_frame(Sympetrum.growth =(d3$Sympetrum[2:19]-d3$Sympetrum[1:18])/d3$Sympetrum[1:18]) %>% mutate(Fipro.dif=diff(d3$Fipronil),Imida.dif=diff(d3$Imidacloprid), Dinotefu.dif=diff(d3$Dinotefuran), Clothia.dif=diff(d3$Clothianidin), Thiametho.dif=diff(d3$Thiamethoxam), Chloran.dif=diff(d3$Chlorantraniliprole), Cartap.dif=diff(d3$Cartap), Benfura.dif=diff(d3$Benfuracarb), Carbosu.dif=diff(d3$Carbosulfan), Neonics.dif=diff(d3$Neonics), Carbamates.dif=diff(d3$Carbamates)) %>% add_column(Year=1994:2011)

model_2 <- glm(Sympetrum.growth~Fipro.dif + Neonics.dif + Cartap.dif + Carbamates.dif, family=gaussian, data = d4[d4$Year<=2004,])
summary(stepAIC(model_2))

## Start: AIC=7.47
## Sympetrum.growth ~ Fipro.dif + Neonics.dif + Cartap.dif + Carbamates.dif
##
## Df Deviance AIC
## <none> 0.42648 7.4658
## - Cartap.dif 1 0.54842 8.2319
## - Neonics.dif 1 0.65724 10.2230
## - Fipro.dif 1 0.98522 14.6760
## - Carbamates.dif 1 2.01447 22.5437

##
## Call:
## glm(formula = Sympetrum.growth ~ Fipro.dif + Neonics.dif + Cartap.dif +
## Carbamates.dif, family = gaussian, data = d4[d4$Year <= 2004,
## ])
##
## Deviance Residuals:
## Min 1Q Median 3Q Max
## -0.51070 -0.06828 0.01120 0.14250 0.23224
##
## Coefficients:
## Estimate Std. Error t value Pr(>|t|)
## (Intercept) -0.04730 0.10688 -0.443 0.67357
## Fipro.dif -0.03346 0.01193 -2.804 0.03101 *
## Neonics.dif 0.02493 0.01384 1.802 0.12166
## Cartap.dif 0.02109 0.01610 1.310 0.23819
## Carbamates.dif 0.05001 0.01058 4.727 0.00324 **
## ---
## Signif. codes: 0 '***' 0.001 '**' 0.01 '*' 0.05 '.' 0.1 ' ' 1
##
## (Dispersion parameter for gaussian family taken to be 0.07108075)
##
## Null deviance: 3.00317 on 10 degrees of freedom
## Residual deviance: 0.42648 on 6 degrees of freedom
## AIC: 7.4658
##
## Number of Fisher Scoring iterations: 2
