# Supplementary material for: Were the sharp declines of dragonfly populations in the 1990s in Japan caused by fipronil and imidacloprid? An analysis of Hill’s causality for the case of Sympetrum frequens
Source: Environ Sci Pollut Res Int. 2018 Oct 20;25(35):35352–64. doi: 10.1007/s11356-018-3440-x (PMC6280840; doi:10.1007/s11356-018-3440-x)
Supplement: Supplementary file 3 — (docx 96.8 kb) [file 11356_2018_3440_MOESM3_ESM.docx]

**Fig. S1** Association between the annual increase of usage ratio of each insecticide or insecticide class and annual growth rate of *Sympetrum frequens* populations in Toyama Prefecture from 1993 to 2004.

**Article title:** Were the sharp declines of dragonfly populations in the 1990s in Japan caused by fipronil and imidacloprid? An analysis of Hill’s causality for the case of *Sympetrum frequens*

**Journal name:** Environmental Science and Pollution Research

**Author names:** Kosuke Nakanishi, Hiroyuki Yokomizo, Takehiko I. Hayashi

**Affiliation and e-mail address of the corresponding author:** National Institute for Environmental Studies, Onogawa 16-2, Tsukuba, Ibaraki 305-8506, Japan; nakanishi.kosuke@nies.go.jp
